# Periprosthetic osteolysis due to metastatic renal cell carcinoma: a case report

**DOI:** 10.1186/1757-1626-1-297

**Published:** 2008-11-05

**Authors:** Asterios Dramis, Aravind S Desai, Tim N Board, Waleed EA Hekal, Jameel R Panezai

**Affiliations:** 1 Department of Lower Limb Arthroplasty, Wrightington Hospital, Appley Bridge, Wigan, WN6 9EP, UK; 2 Department of Orthopaedics, Bassetlaw District General Hospital, Worksop, S81 0BD, UK

## Abstract

Failure of total hip arthroplasty through septic or aseptic loosening, periprosthetic fracture, or recurrent dislocation is well recognized and understood. We present an unusual cause of failure of total hip replacement which occurred on a 79 year old gentleman: that of prosthetic loosening secondary to malignant infiltration around components. Our aim is to highlight the fact that malignant infiltration should be considered as part of the differential diagnosis in aseptic and septic loosening of prosthetic implants.

## Case presentation

**Figure 1:**
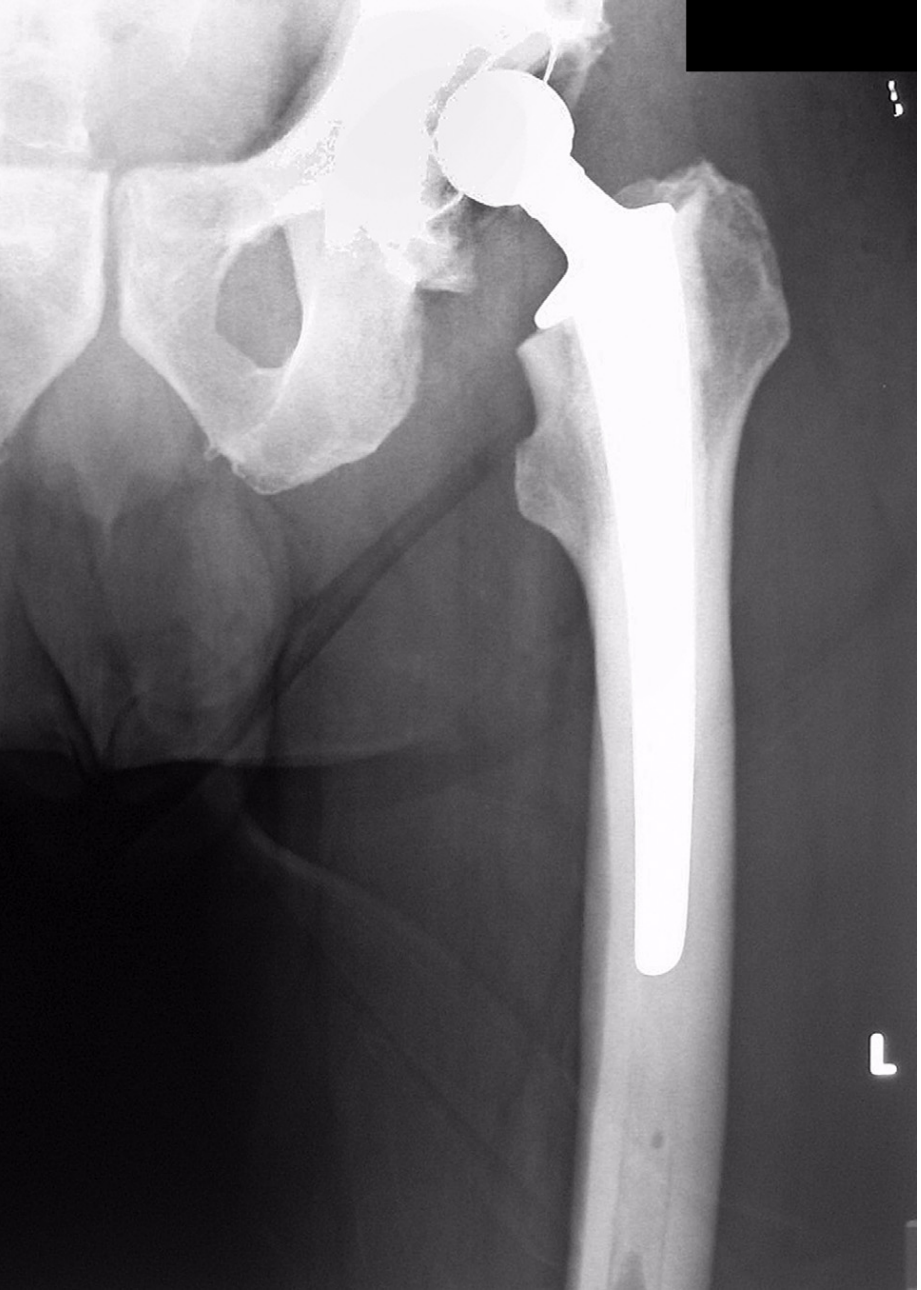
Postoperative radiograph of the left hip at 5 days showing the total hip replacement.

**Figure 2:**
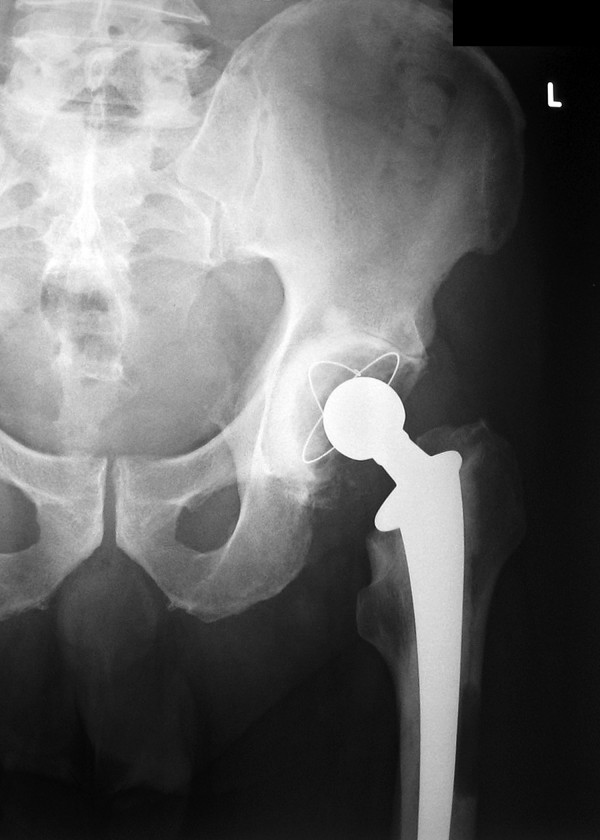
Postoperative radiograph of the left hip at 6 months showing an osteolytic lesion in Gruen zones 2 & 3 at the femoral stem.

A 79-year-old Caucasian gentleman, with no history of any malignancy, underwent a left cemented total hip replacement (THR) for osteoarthritis and was symptom free post-operatively (Figure 1). At 6 months he complained of pain in the left groin and thigh. Examination revealed painless hip movements. Radiographs of the left hip showed an osteolytic lesion in Gruen zones 2 and 3 (Figure 2). Routine blood investigations showed an erythrocyte sedimentation rate of 90 mm/hr and C- reactive protein of 50 mg/dl. Aspiration of left hip did not reveal any organisms and a bone scan showed increased uptake of on the left femoral shaft, right scapula and the first lumbar vertebra. Bone chemistry, tumour markers, renal and liver assay were normal. An open biopsy showed erosion of the lateral cortex associated with friable soft tissue mass and profuse bleeding. Histopathological report showed a classical case of renal cell carcinoma. Further to this, a computed tomographic (CT) scan of the abdomen and chest revealed multiple nodules in lung fields, multiple nodules in liver, a mass on each kidney consistent with renal cell carcinoma and multiple skeletal lytic lesions.

The patient was then referred to the oncologist for palliative treatment and died 6 months later.

## Discussion

Metastatic spread to a joint replacement is exceptional. To date, there have been very few reports in the literature documenting periprosthetic metastatic disease as a mode of failure in total hip and knee arthroplasty. These consisted of a non-Hodgkin lymphoma [1], an immunoblastic lymphoma [2], bronchogenic carcinoma [2-4], gastric carcinoma [5,2], prostatic and breast carcinoma [6], renal cell carcinoma [6,7], metastatic thyroid [8], ovarian and hepatocellular carcinoma [9].

A metastatic lesion in a patient who has undergone prior THR may appear as solitary periprosthetic lucency and may be misinterpreted as aseptic periprosthetic osteolysis. Aseptic osteolysis may be extensive in size, appears as endosteal scalloping and does not usually invade the outer cortex. Single lesions that involve the whole cortex, appear soon after implantation, are painful and progress rapidly are not typical of aseptic osteolysis and should raise a suspicion of malignancy.

Furthermore, according to study done by Mohler et al [10], early loosening of femoral component at cement prosthesis interface occurs at Gruen zones 1 and 2, and any lucent areas in zones 3 and 4 should be suspicious of metastatic lesions. In our case, the patient presented soon after the total hip replacement with pain and radiographic signs of an osteolytic lesion invading the outer cortex.

One should maintain a high index of suspicion and consider metastatic disease as a differential diagnosis in cases of aseptic loosening, particularly when there is rapid progression of symptoms, the history is atypical, the patient has a history of malignant disease and the osteolytic lesion involves the outer cortex. If these features are present, the lesion should be biopsied and appropriate radiological and haematological investigations should be considered.

## Consent

"Written informed consent was obtained from the patients' next of kin for publication of this case report and accompanying images. A copy of the written consent is available for review by the Editor-in-Chief of this journal."

## Competing interests

The authors declare that they have no competing interests.

## Authors' contributions

AD and ASD wrote the draft of the manuscript and performed the literature search; TNB revised the manuscript for intellectual content; JRP and WEAH performed the surgical procedure.
